# Development of a Vaccine Candidate Based on Surface-Displayed Particles of *Mycobacterium tuberculosis* from the MTB39A Protein

**DOI:** 10.3390/ijms26020797

**Published:** 2025-01-18

**Authors:** Pu Wang, Gang Zhang, Lingling Jiang, Sinong Zhang, Weifeng Gao, Zhiwei Wu, Yong Li

**Affiliations:** 1 Key Laboratory of Ministry of Education for Protection and Utilization of Special Biological Resources in Western China, School of Life Sciences, Ningxia University, Yinchuan 750021, China; wangpu1175@163.com (P.W.); fuzang123@163.com (G.Z.); jianglingling0512@163.com (L.J.); sinongzhang@nxu.edu.cn (S.Z.); stephanie.wf@163.com (W.G.); 2 Center for Public Health Research, Medical School, Nanjing University, Nanjing 210023, China

**Keywords:** MTB39A, *Mycobacterium tuberculosis*, baculovirus expression system, nanoparticles, vaccine

## Abstract

Tuberculosis (TB), a human and animal disease caused by *Mycobacterium tuberculosis* (*M.tb*), has the highest global mortality rate after coronavirus disease 2019 (COVID-19) and poses a major public health threat globally. Since 1890, vaccine candidates for various forms of TB have been developed for different age groups, but these vaccine candidates have not provided intended protection in adolescents and adults in clinical trials. To help prevent and control the spread of TB, the development of a safe and effective TB vaccine is imperative. The MTB39A protein and the molecular adjuvant MTB32C protein were expressed by an insect-baculovirus expression system, and the recombinant baculovirus surface-displayed particles were evaluated for their immunogenicity in BALB/c mice and calves. The results showed that the rvAc-71CA/rvAc-MTB39A recombinant baculovirus surface-displayed particles exhibited good immunogenicity in mice and calves and could be further developed as potential candidates.

## 1. Introduction

Tuberculosis (TB) is a chronic infectious disease that primarily affects the lungs (pulmonary TB) but can also manifest in other parts of the body (extrapulmonary TB) [1]. When humans and cows with TB cough, droplets are discharged into the air, which can be inhaled by healthy people and cows to cause infection. According to the Global TB Report 2023, there are approximately 10 million new cases each year, and approximately one in four people worldwide have latent TB infection (LTBI), of which 5–10% will develop active TB, resulting in 1.3 million deaths. The incidence of human tuberculosis caused by *Mycobacterium bovis* accounts for 5–10% [2]. Although bovine tuberculosis has been controlled in many developed countries, it still threatens people’s health in many developing countries, posing a major public threat in many parts of the world [3].

Vaccination remains the primary means of preventing and controlling TB. Bacillus Calmette–Guerin (BCG) is the most time-tested and widely used TB vaccine and remains the only licensed TB vaccine; thus, BCG provides protection for kids, but its efficacy has been reduced in adults [4]. However, BCG has significant limitations, especially being age-dependent, that is, the inability to provide adequate protection against TB for adolescents, adults, and adult cattle [5,6]. Subunit vaccine candidates are designed to augment BCG-triggered responses and mainly include viral vector or recombinant protein-adjuvanted vaccines, of which recombinant protein-adjuvanted subunit vaccines are more attractive due to their excellent safety profile, well-defined molecular composition, and absence of an immune response generated by the body against the vector [7].

Members of a specific glycine-rich gene family, the PE/PPE gene family, are encoded in the *M.tb* genome, accounting for approximately 10% of the genomic coding region and including a total of 168 genes. The members of this family play key roles in the virulence, subcellular localization, and immune escape of *M.tb* and the host cell fate [8]. Among these members, MTB39A (PPE18), encoded by the Rv1196 gene, is able to inhibit macrophage-mediated presentation of MHC class II antigens in a dose-dependent manner [9,10,11]. Previous studies have shown that the C-terminal structural domain of MTB32A (MTB32C), which is recognized by IFN-γ-secreting CD_8_^+^ T cells [12], produces strong cellular responses in pre- and post-exposure mouse models of *M.tb*, making it an ideal candidate vaccine antigen. *M.tb* infections are a major source of anti-infective immunity, and CD_4_^+^ T cells—particularly the Th1 and Th17 subtypes—are essential for this defense. Th1 cells secrete IFN-γ (interferon-γ) and other cytokines that stimulate macrophages and aid in the destruction of intracellular pathogens, whereas Th17 cells aid in the recruitment of neutrophils and fortify mucosal defense by secreting IL-17 [13]. Ag85A and ESAT-6, two *M.tb* components, have been proven in vaccine experiments to be efficacious in eliciting CD4 T cell responses, namely Th1 and Th17 responses. In order to enhance protection against *M.tb*, Ag85A and ESAT-6 are frequently utilized in vaccine formulation to trigger a particular CD4 T cell immunological response [14].

Insect baculovirus expression systems can express some simple glycosylated proteins at high levels, are simple to operate, and can be used for vaccine development, particularly regarding adjuvant activity and safety. In this study, the MTB39A protein from *M.tb* was successfully expressed and localized using the baculovirus surface display system, and the resulting recombinant baculovirus surface-displayed particles were evaluated in mice and calves. The results indicated that the baculovirus surface display system can be applied as an effective method for the development of *M.tb* subunit vaccines.

## 2. Results

### 2.1. Construction and Characterization of MTB32C-MTB39A Recombinant Vector

**Figure 1:**
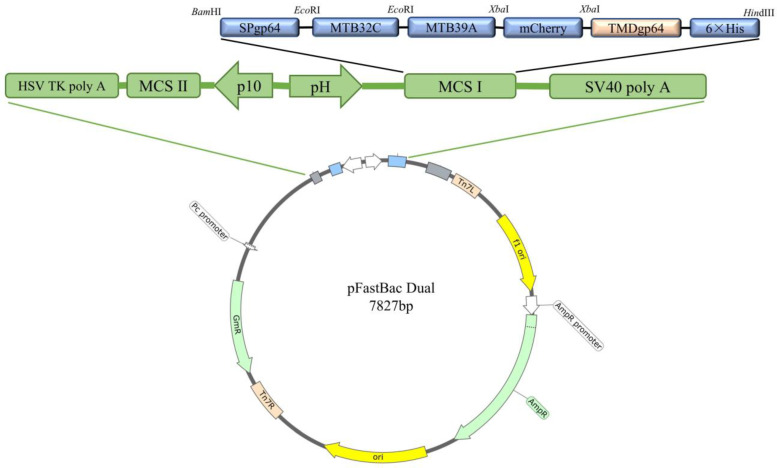
Schematic presentation of the recombinant vector pFastBac dual-71CA-mCherry.

**Figure 2:**
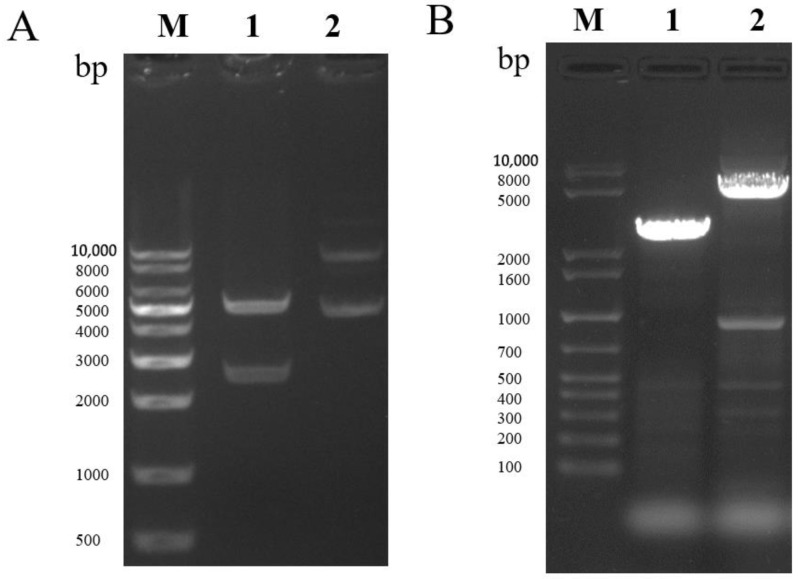
(**A**) Double digest of the recombinant vector pFastBac dual-71CA-mCherry. Lane 1 shows the double digest *Bam*HI-*Hin*dIII (2589–5238 bp), and Lane 2 shows the recombinant vector plasmid. (**B**) PCR identification of recombinant bacmid-71CA-mCherry. Lane 1 shows the pFastBac dual empty vector, and Lane 2 shows Bacmid-71CA-mCherry.

Expression plasmid pFastBac dual-MTB32C-MTB39A-mCherry (pFastBac dual-71CA-mCherry, Figure 1) was constructed, and MTB32C and MTB39A were identified by gene synthesis followed by sequencing. PCR was performed to validate the bacmid using the M13 universal primer (Figure 2A,B), and full-length gels are presented in Supplementary Figure S1. The recombinant baculovirus display particles were named as rvAc-71CA-mCherry, and the supernatant of the infected cells was collected for virus preparation. The purified and concentrated virus (10^7^ PFU/mL) was stored at −80 °C.

### 2.2. Localization of Antigenic Expression in Recombinant Baculovirus Surface Display Particles rvAc-71CA-mCherry

**Figure 3:**
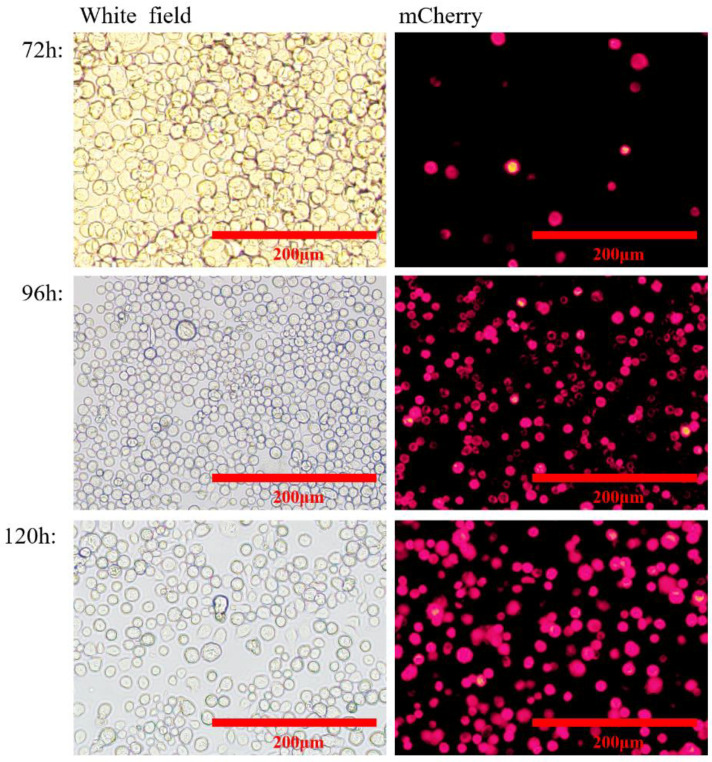
Visualization of rvAc-71CA-mCherry-infected Sf9 cells through mCherry fluorescence.

**Figure 4:**
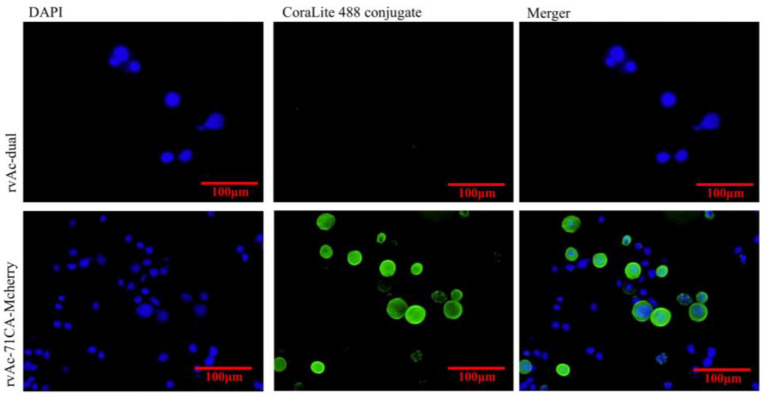
Indirect immunofluorescence of recombinant virus- and wild-type virus-infected cells.

**Figure 5:**
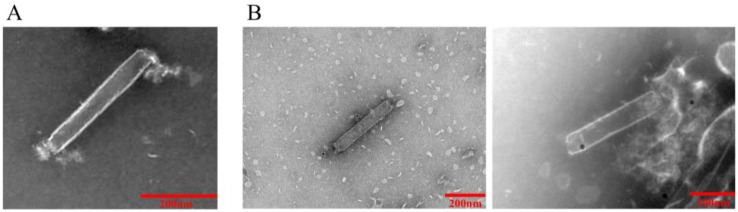
Electron micrograph of recombinant baculovirus rvAc-71CA-mCherry. (**A**) rvAc-dual was incubated with anti-mCherry monoclonal antibody. (**B**) rvAc-71CA-mCherry was incubated with anti-mCherry monoclonal antibody.

At 72, 96, and 120 h after transfection, the expression of red fluorescent protein was assessed by immunofluorescence identification and transmission electron microscopy. The results showed that the nuclei of both rvAc-71CA-mCherry and wild-type viruses showed blue fluorescence and could be stained by DAPI, while only cells infected with rvAc-71CA-mCherry produced green fluorescence signals. It was demonstrated that the protein expressed by cells infected with rvAc-71CA-mCherry could successfully locate on the surface of the cell membrane (Figure 3 and Figure 4). Immunocolloidal gold labeling of recombinant baculovirus revealed that the morphology of the fluorescent recombinant virus was basically similar to that of the wild-type baculovirus; both were rod-shaped, and the particle size was about 60 nm × 300 nm. On the surface of recombinant viruses incubated with mCherry, colloidal gold particles with a diameter of about 10 nm were gathered in the capsule region, but no colloidal gold particles were attached to the surface of wild-type viruses. The fluorescent protein that proves the success of the recombinant virus is displayed on its surface (Figure 5).

### 2.3. rvAc-71CA/rvAc-MTB39A Induces Immune Responses in Mice

**Figure 6:**
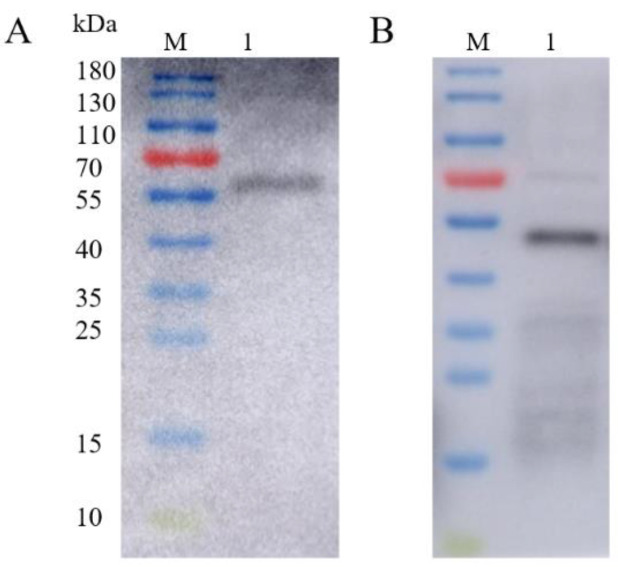
Western blot identification of recombinant baculovirus surface-displayed particles. (**A**) M: 10–180 kDa; Lane 1: Western blot identification of rvAc-71CA (63.5 kDa). (**B**) M: 10–180 kDa; Lane 1: Western blot identification of rvAc-MTB39A (50.5 kDa).

**Figure 7:**
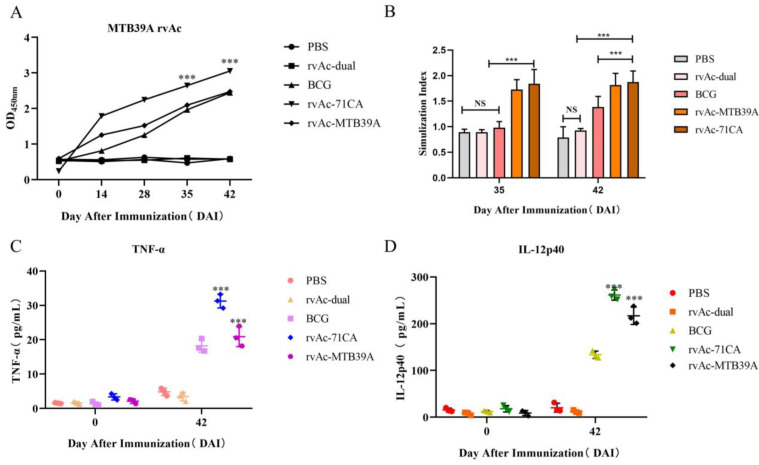
Analysis of immunogenicity in mice. (**A**) Analysis of the IgG response induced by the MTB39A recombinant protein in mice measured by indirect ELISA. The *y*-axis indicates the optical density (OD_450_) values of serum samples collected from each group at 0, 14, 28, 35, and 42 DAI. *** *p* < 0.001, significantly different from the PBS, rvAc-dual, and BCG groups (Bonferroni test). (**B**) Results of the lymphocyte proliferation assay. The *y*-axis represents the stimulation of splenic lymphocyte samples collected at 35 and 42 DAI. ns, no significant difference; *** *p* < 0.001, significantly different (Bonferroni test). (**C**,**D**) Quantitative analysis of the IL-12p40 and TNF-α levels in the serum of immunized mice. All analyses were performed in triplicate, and the error bars show the standard deviations (SDs).

The fluorescent protein and/or MTB32C protein was excised to obtain the corresponding recombinant baculovirus-displayed nanoparticles rvAc-71CA/rvAc-MTB39A, and the expression of viral antigens was detected by Western blotting with an anti-6×His (Figure 6), and full-length blots are presented in Supplementary Figure S2. To assess the humoral immune response induced by both nanoparticles in mice, the IgG levels were measured by indirect enzyme-linked immunosorbent assay (ELISA), and the results are shown in Figure 7A. At 35 and 42 DAI, the serum anti-MTB39A IgG levels of the rvAc-71CA/rvAc-MTB39A group were significantly higher (*p* < 0.001) than those of the two negative control and BCG groups.

At 35 and 42 DAI, the splenocyte stimulation index was significantly higher in the rvAc-71CA/rvAc-MTB39A group than in the PBS, rvAc-dual, and BCG groups (*p* < 0.001; Figure 7B). The stimulation level observed with knife bean protein A (positive control for lymphocyte proliferation assay) was considered effective. The IL-12p40 and TNF-α levels in mouse serum were determined at 0 and 42 DAI. The rvAc-71CA/rvAc-MTB39A group exhibited significantly higher levels of IL-12p40 and TNF-α than the other three groups (*p* < 0.001; Figure 7C,D). The serum IL-12p40 and TNF-α levels of mice immunized with recombinant baculovirus surface-displayed nanoparticles of rvAc-71CA at 42 DAI were 261.27 ± 6.32 pg/mL and 31.25 ± 1.15 pg/mL, respectively, and the serum IL-12p40 and TNF-α levels of mice immunized with recombinant baculovirus surface-displayed nanoparticles of rvAc-MTB39A at 42 DAI were 216.93 ± 10.74 pg/mL and 20.89 ± 1.68 pg/mL, respectively. The results showed that the recombinant baculovirus induced cellular immunity in mice.

### 2.4. Evaluation of rvAc-71CA/rvAc-MTB39A Nanoparticle Vaccine-Activated T-Cell Responses and Safety in Mice

**Figure 8:**
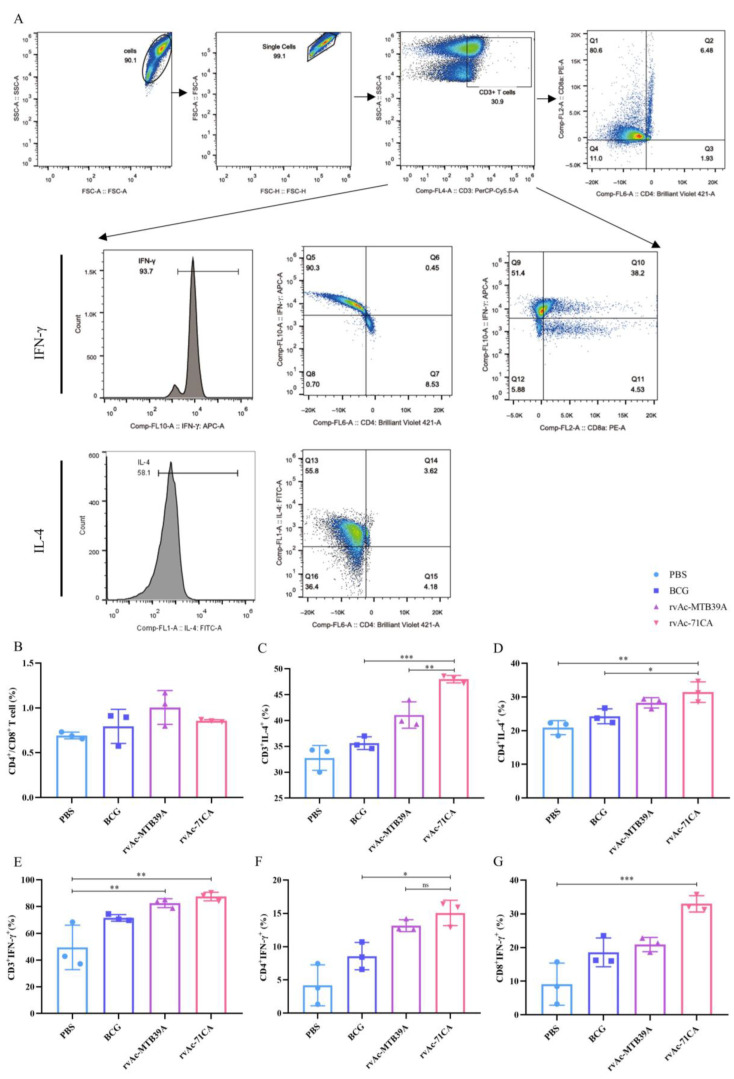

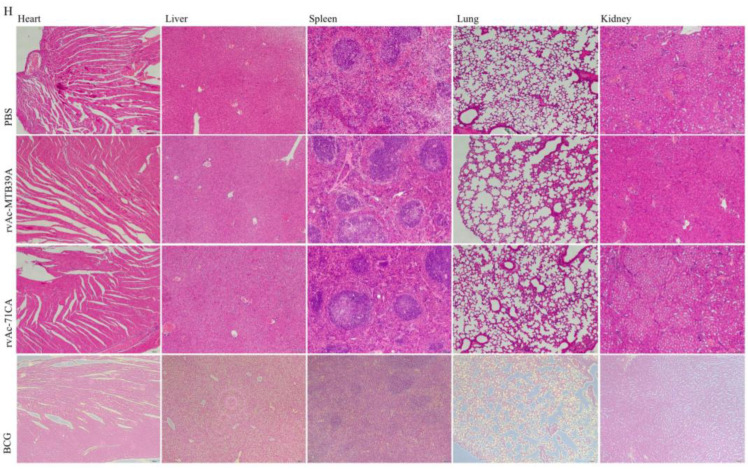
T cell proliferation and safety evaluation of mice immunized with nanoparticle vaccines. (**A**) Flow cytometer gate strategy. (**B**) Percentage of CD4^+^/CD8^+^ T cells in splenic lymphocytes of immunized mice, (**C**) CD3^+^IL-4^+^ T cells, (**D**) CD4^+^IL-4^+^, (**E**) CD3^+^IFN-γ^+^, (**F**) CD4^+^IFN-γ^+^, and (**G**) CD8^+^IFN-γ^+^. (**H**) Haematoxylin–eosin (HE) staining of tissue sections of major organs (10×, *n* = 3). The data are shown as the means ± SDs and analyzed by two-way ANOVA using the Bonferroni test. ns, not significant; * *p* < 0.05; ** *p* < 0.01; *** *p* < 0.001.

IFN-γ and IL-4 will induce the proliferation of CD4^+^ and CD8^+^ T cells, which mediates adaptive immunity to eradicate the infected cells. To examine the expression of IFN-γ and IL-4, we isolated lymphocytes from the spleens of immunized mice. The rvAc-MTB39A group had a higher CD4^+^/CD8^+^ ratio than rvAc-71CA, suggesting that the rvAc-MTB39A vaccine group elicited immunity primarily from CD4+ T cells, and rvAc-71CA elicited immunity primarily from CD8^+^ T cells (Figure 8A,B). As shown in Figure 8C–G, the rvAc-71CA immunized group secreted higher levels of IFN-γ^+^ and IL-4^+^ cells, inducing stronger cellular immune responses by T cells.

The safety of a vaccine plays a very important role in its clinical application. We examined the H&E-stained sections of the heart, liver, spleen, lungs, and kidneys of each group at 42 days and found no nodules or necrosis, among other features. Some tissues were then collected for H&E staining, and the results of microscopic examination are shown in the figure (Figure 8H). Tissue sections of the selected organs did not show any obvious pathological changes, such as congestion, bruising, necrosis, and inflammatory exudation, and had good biocompatibility.

### 2.5. rvAc-71CA/rvAc-MTB39A Induced Immune Response in Calves

**Figure 9:**
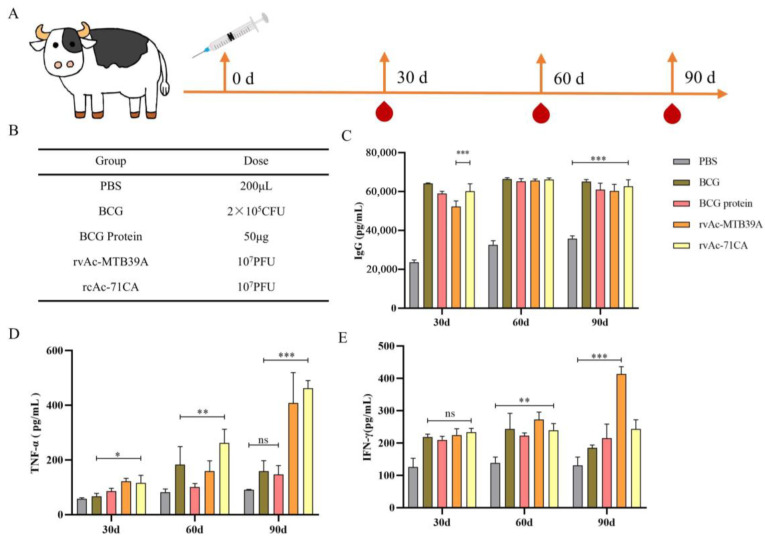
Immunogenicity analysis of calves. (**A**) Calf immunization strategy; (**B**) calf immunization groups and doses; (**C**) IgG immune response; (**D**) quantitative analysis of cytokine TNF-α levels; and (**E**) quantitative analysis of cytokine IFN-γ levels. ns, not significant; * *p* < 0.05; ** *p* < 0.01; *** *p* < 0.001, significant difference (Bonferroni test).

As a zoonotic pathogen, TB infection of cattle poses a dire challenge not only to the animal husbandry industry but also to human health. Therefore, we also investigated the immunogenicity of the recombinant subunit vaccine in calves and analyzed the cytokine levels in the peripheral blood of calves (Figure 9A,B). After 30 d of immunization, calves in the rvAc-71CA/rvAc-MTB39A group were detected in vivo with IgG levels of (60,098.53 ± 2224.81 pg/mL, 52,269.23 ± 1674.35 pg/mL), 60 d IgG levels of (61,182.76 ± 447.33 pg/mL, 60,654.567 ± 430.86 pg/mL), and 90d IgG levels were (62,625.83 ± 3393.89 pg/mL, 60,279.03 ± 3417.33 pg/mL), which were not significantly different from BCG and BCG protein. The levels of TNF-α (462.42 ± 27.84 pg/mL, 407.73 ± 111.73 pg/mL) and IFN-γ (242.92 ± 29.15 pg/mL, 413.09 ± 22.82 pg/mL) in the peripheral blood were significantly higher than those in the PBS group (*p* < 0.001, Figure 9C–E), indicating that the rvAc-71CA subunit vaccine elicited significant humoral immune response and the Th1-type cellular immune response in calves.

## 3. Discussion

As the number of TB cases and the comorbidity of TB and HIV increase, the only approved vaccine, BCG, is insufficient to achieve TB elimination in this century [15,16], and new prevention and control measures are urgently needed [17], among which the development of new and effective TB vaccines is an important means of preventing TB [18]. In this way, the immune system of *M.tb*-infected individuals can establish immune homeostasis, inhibit immune damage, enhance immunity, or clear *M.tb* [19]. M72/AS01E is a tuberculosis vaccine candidate designed to prevent the progression of latent tuberculosis infection to active tuberculosis. The vaccine consists of the recombinant protein antigen M72 (MTB39A and MTB32A) together with the AS01E adjuvant system, which is an immune-enhancing adjuvant. Clinical trials have demonstrated that M72/AS01E shows some protection in healthy adults as well as in people with latent TB infection, particularly in preventing TB progression. The vaccine offers a potential new tool in the global response to the TB epidemic, but its long-term efficacy and broad applicability remain to be validated in further clinical studies. The first vaccine candidate with protective efficacy against tuberculosis in clinical phase IIb trials was the modified Ankara vaccine (MVA), a viral vector vaccine expressing *M.tb* Ag85 complex antigens; however, the T-cell activation and elevation of the blood monocyte/lymphocyte ratios induced by this vaccine increase the risk of developing TB, and vaccination failed to produce any protective effect [20,21]. Subunit vaccines benefit from having a novel adjuvant with potent T-cell stimulation ability and no harmful side effects, and one such vaccine was designed to enhance the BCG-triggered response [22,23]. Among these, subunit vaccines that specifically target important *Mycobacterium* TB antigens like Ag85A/B and ESAT-6 can elicit particular immune responses. These vaccinations are intended to be administered in conjunction with BCG in order to boost immunological effectiveness and give recipients of the BCG vaccination extra protection.

However, in this study, BCG was not used for initial immunization, and the subunit vaccine was used for booster immunization. We hope that the prepared subunit vaccine can achieve the initial immunization effect of replacing BCG, with a view to providing an experimental basis for the development of a new type of tuberculosis vaccine. Recent years have seen some advancements in the study of subunit vaccines; these include the H56 and ID93 vaccines, which have shown promise in preclinical and early-stage clinical studies for inducing a robust T cell response [24]. The subunit vaccine produced in this study will eventually integrate protection tests and immunogenicity data to further measure the vaccine’s protective impact. This will help to guarantee that the vaccination is effective in preventing tuberculosis and will allow us to better assess the vaccine’s clinical application potential [25].Baculovirus expression vector systems (BEVSs) have the advantages of a high level of exogenous gene expression, high biosafety, and a short replication time [26,27,28]. BEVSs have been widely used in biopharmaceuticals, genetic engineering, and vaccine research and production, and the production of HPV and influenza virus vaccines using this system has shown good results [29,30].

MTB39A protein and MTB32A protein can enhance phagocytic activity, and the C-terminal structural domain MTB32C can produce a strong CD8 T-cell immune response, which are candidate vaccine antigens [31]. In this study, we chose to use the baculovirus expression system with the PE/PPE protein family members, MTB39A protein and MTB32C protein, in order to achieve similar or better immunization effects than the M72 vaccine. And to provide a reference for vaccines prepared by such systems.

In this study, the *M.tb* protein MTB32C-MTB39A(71CA) was successfully expressed by a BEVS. The expression of nanoparticles labelled with mCherry could be directly observed under an inverted microscope (Figure 3), demonstrating that they were displayed on the plasma membrane of Sf9 cells (Figure 4) and on the vesicle membrane of the virus (Figure 5). These results indicate that the recombinant baculovirus displaying the mCherry-tagged MTB39A protein was correctly assembled and able to infect Sf9 cells. The immunogenicity of the recombinant protein MTB39A was verified in both mice and calves. In mouse immunization experiments, rvAc-71CA induced a robust IgG response in the rvAc-dual and rvAc-MTB39A groups at 35 and 42 DAI. This result suggests that rvAc-71CA can induce the development of strong humoral immunity against the MTB39A antigen in mice (Figure 7A). The lower antibody levels in the rvAc-MTB39A group compared with the rvAc-71CA group possibly occurred because the MTB32C protein enhances the immunogenicity of the MTB39A protein.

In cellular immunity, both CD_4_^+^ T and CD_8_^+^ T cells are central to the defense against *M. tb* infection, and their content and CD_4_^+^ T/CD_8_^+^ T cell ratios are commonly used to measure the state of cellular immunity in the body [32]. Lymphocyte proliferation and cytokine production after antigenic stimulation are also important indicators of immune effects. The results of the lymphocyte proliferation assay and flow cytometry showed that rvAc-71CA/rvAc-MTB39A induced strong cellular immunity against the MTB39A antigen (Figure 7B) and increased the serum levels of IL-12p40 and TNF-α in BALB/c mice (Figure 7C,D). The expression of CD4^+^IFN-γ^+^, CD8^+^IFN-γ^+^, and CD4^+^IL-4^+^ T cells in the rvAc-71CA immunized group was higher than that in the rv-MTB39A group, suggesting that the MTB32C protein elicits a strong CD8^+^ T-cell immune response, which is able to participate in the killing of infected cells (Figure 8A–G) and that the nanoparticle vaccine exhibited good biocompatibility (Figure 8H). In the calf immunization assay (Figure 9A,B), rvAc-71CA/rvAc-MTB39A significantly increased the levels of serum antibody IgG (Figure 9C) and the cytokines TNF-α and IFN-γ (Figure 9D,E), which resulted in the host being able to resist *M.tb* [33].

## 4. Materials and Methods

### 4.1. Construction of the MTB32C-MTB39A Gene and Expression Vector

Based on studies of the MTB39A protein and MTB32A protein, we selected the gene sequence corresponding to the MTB39A protein of M.tb H37Rv (GenBank ID: NC_000962.3) and intercepted the MTB32A sequence (GenBank ID: MTCI418B.07) of the 396-bp C segment (MTB32C). The MTB39A and MTB32C gene sequences were designed to encode the fusion protein MTB32C-MTB39A with a His tag at the C-terminus, referred to as 71CA. mCherry was used as the protein tag for visualization in assays. The sequence SPgp64 (GenBank AFO10080.1) was inserted upstream of the target protein. The downstream tag protein was inserted into TMDgp64 (GenBank CAA24524.1). The fusion gene fragment SPgp64-71CA-mCherry-TMDgp64-6×His was then inserted into the pFastBac dual vector to generate pFastBac dual-71CA-mCherry (Table 1).

### 4.2. Culture of Grassland Nightshade Moth (Sf9) Cells and Amplification of Recombinant Baculovirus

Insect Sf9 cells were cultured at 27 °C and used for gene expression. The recombinant plasmid pFastBac dual-71CA-mCherry and the plasmid pFastBac dual (negative control) were transformed into DH10 Bac, and the positive colonies obtained were amplified with M13 universal primers. The positive bacilli were named Bacmid-71CA-mCherry, and the negative bacilli were named Bacmid-dual. Sf9 cells in good growth condition were spread into 6-well plates, ensuring that cell confluence was around 80%. To prepare the transfection mixture, 95 μL of Sf900 medium and 5 μL of TransIT^®^-LT1 Transfection Reagent (Mirus) were added [34]. Into a 1.5 mL ep tube, 1 μg of Bacmid was added, the volume was made up to 100 μL with the medium, and the sample was incubated for 5 min at room temperature. Finally, the diluted Bacmid was gently added into the transfection reagent, blown and mixed, and left for 20 min at room temperature. The diluted Bacmid was gently added to the transfection reagent; it was blown and mixed and kept at room temperature for 20 min. Then, 200 μL of transfection mixture was placed drop by drop into each well of pre-layered Sf9 cells and cultivated in the cell culture incubator at 27 °C. The viruses produced by the transfected virus were of the P0 generation.

The rvAc-71CA/rvAc-MTB39A viruses were implanted into suspension-cultured Sf9 cells at a density of roughly 2 × 10^6^ cells/mL with MOI = 0.1. Following 72 h of infection, the suspension was collected by centrifugation at 2500 rpm for 5 min at 4 °C, cell debris was removed by centrifugation at 10,000 rpm for 30 min, and virus particles were recovered by ultracentrifugation at 35,000 rpm for 2 h. The virus particles were recovered via ultracentrifugation at 35,000 rpm. The virus particles were added to a gradient sucrose solution containing 20–40–60% sucrose, ultracentrifuged at 35,000 rpm for 2 h at 4 °C, and the turbid zone between the 40–60% sucrose solution was collected, ultracentrifuged again to remove sucrose, then solved with PBS and stored at -80 °C.

### 4.3. Indirect Immunofluorescence and Immunocolloidal Gold Electron Microscopy

To visualize the location of fusion protein 71CA in infected Sf9 cells, recombinant baculovirus surface-displayed particles rvAc-71CA-mCherry and rvAc-dual (MOI of infection plural = 0.5) were infected into Sf9 cells until cytopathic lesions appeared, supernatants were discarded, and cells were fixed with 4% paraformaldehyde (Solarbio, Beijing, China), 0.2% Triton X-100 (Solarbio, China), Permeabilization was performed, 5% BSA blocking solution was blocked for 1 h, mouse anti-His monoclonal antibody (1:500 dilution; Abcam, Shanghai, China) was incubated overnight, PBS was washed 3 times, CoraLite 488 coupled goat anti-mouse IgG (secondary antibody, 1:500 dilution; Proteintech, Wuhan, China) was incubated, and nuclei were stained with DAPI. Fluorescence images were taken with a laser confocal microscope (Leica TCS SPE, Wetzlar, Germany).

To determine the location of the fusion protein 71CA in the baculovirus envelope, rvAc-dual and rvAc-71CA-mCherry were adsorbed in a 200-mesh copper mesh for 5 min, air-dried, washed with 0.01 mol/L PBS for 2 min, blocked with 1% BSA for 1 h, and incubated with a mouse anti-mCherry monoclonal antibody (1:1000 dilution) for 2 h at room temperature (Abmart, Shanghai, China). The secondary antibody, protein-A-gold coupled with colloidal gold particles, was incubated at room temperature for 1 h. After washing, the sample-loaded copper mesh was naturally dried, dyed with 3% phosphotungstic acid solution for 5 min, and photographed at 80 kV in a transmission electron microscope (Hitachi HC-1, Tokyo, Japan).

### 4.4. Vector and Western Blot Identification for Animal Immunization Experiments

To obtain proteins without a fluorescent tag and MTB32C, two vectors, pFastBac dual-71CA (with the tag protein mCherry removed) and pFastBac dual-MTB39A (with the mCherry and MTB32C proteins removed), were produced. The recombinant baculovirus displaying rvAc-71CA/rvAc-MTB39A was obtained using the method described above, and rvAc-dual served as a negative control. The baculovirus rvAc-71CA/rvAc-MTB39A was identified by protein blotting using the following antibodies: mouse anti-His monoclonal antibody (1:5000, Abcam) and HRP-conjugated AffiniPure goat anti-mouse IgG (H + L) (1:5000, Proteintech).

### 4.5. Mouse Vaccination

Seventy-five 6–8-week-old healthy female BALB/c mice were purchased from Beijing Vitonda Biotechnology Co., Ltd., Beijing, China (certificate of conformity No. SCXK Jing 2021-0006) and randomly divided into five groups of 15 mice each (Table 2). Immunization was performed by subcutaneous injection at 2-week intervals, and the BCG and experimental dose was based on relevant studies [35,36]. Serum samples were collected at 0, 14, 28, 35, and 42 days after immunization (DAI), and three mice in each group were sacrificed at 35 and 42 DAI. Spleens were isolated for lymphocyte proliferation assays, and mouse experiments were performed in accordance with the recommendations of the Experimental Ethics Committee of the Experimental Animal Center of Ningxia Medical University (IACUC-NYLAC-2021-126).

### 4.6. Analysis of the Serum IgG Antibody Levels

The enzyme plate was coated with MTB39A protein at a concentration of 1 μg/mL (0.05 mol/L carbonate buffer, pH 9.6) overnight at 4 °C, incubated with 5%BSA at 37 °C for 2h, and then the added serum samples were collected by 0, 14, 28, 35, and 42 DAI (1:100 dilution), incubated for 1h, and washed with PBST 3 times. Then, the samples were incubated with enzyme-labeled goat anti-mouse IgG secondary antibody for 1h (1:100 dilution; Proteintech). Finally, the reaction was stopped using 2M H_2_SO_4_ after 30min of TMB color (Solarbio, China) development solution. All the samples were analyzed in triplicate, and the absorbance was determined with a microplate reader (EnSpire, Waltham, MA, USA) at OD450 nm.

### 4.7. Lymphocyte Proliferation Test

rvAc-71CA/rvAc-MTB39A, 100 μL of RPMI-1640 (negative control, Gibco, NY, USA), and knife bean protein A (5 μg/mL; Solarbio, Beijing, China, positive control) were used as stimulants at 10^6^ PFUs/mL. Splenic lymphocyte proliferation was detected using the MTT assay (5 mg/mL; Solarbio Beijing, China) by determining the OD490 nm. The cellular immune effect was reflected by the stimulation index (SI) of the splenic lymphocyte proliferation assay: SI = mean OD490 value of the stimulated wells/mean OD490 value of the negative control wells.

### 4.8. Flow Cytometry

Splenocytes were isolated 42 days after immunization, inoculated into 6-well plates at 1 × 10^6^ cells per well, and stimulated in vitro with 5 μg/mL of rvAc-71CA and rvAc-MTB39A for 12 h. The cells were centrifuged at 1000 rpm for 5min, collected, and then used for surface staining (Proteintech, Wuhan, China): CoraLite^®^Plus 405 Anti-Mouse CD4 (GK1.5, Proteintech, Wuhan, China)), PE Anti-Mouse CD3 (17A2, Proteintech, Wuhan, China)), and CoraLite^®^Plus 705 Anti-Mouse CD8a (53-6.7, Proteintech, Wuhan, China)). After permeabilization of the cells with complete surface staining, the intracellular cytokine staining antibodies used were CoraLite^®^Plus 488 Anti-Mouse IL-4 (11B11, Proteintech, Wuhan, China)) and CoraLite^®^Plus 647 Anti-Mouse IFN-γ (XMG1.2, Proteintech, Wuhan, China)). The cells were then detected with a SONY MA900 and analyzed using FlowJo 10.0 (Tree Star Inc., Ashland, OR, USA) software.

### 4.9. H&E Staining

The hearts, livers, spleens, lungs, and kidneys of mice in each group at 42 d were taken, fixed in 4% paraformaldehyde, and after paraffin sectioning, the nuclei of the cells were stained with hematoxylin, the cytoplasm was stained with eosin, and finally dehydrated and sealed, and under an inverted microscope, the images were captured.

### 4.10. Immunization Strategy and Cytokine Detection in Calves

Fifteen calves at 21 days of age were randomly divided into five groups of three calves each. The first immunization was administered via a subcutaneous injection, and the doses of the commercial vaccine BCG and cleaved whole BCG protein (whole bacterial lysate) were based on relevant research literature. The recombinant baculovirus group, the BCG, and the BCG protein all require only one injection and no booster immunization. Blood was collected intravenously 30, 60, and 90 days after the initial immunization, and serum was collected by centrifugation. The serum antibody levels of IgG and the levels of the cytokines TNF-α and IFN-γ were detected by indirect ELISA. The calf experiments were performed according to the recommendations of the Experimental Ethics Committee of the Experimental Animal Center of Ningxia University.

### 4.11. Statistical Analysis

The statistical analyses were performed using GraphPad Prism^®^ Version 6 (GraphPad Software, San Diego, CA, USA) software. The immunoreactivity of the groups was compared by Bonferroni’s test. The data are expressed as the means ± SDs, and *p* < 0.05 was considered to indicate a significant difference.

## 5. Conclusions

This report describes the production, identification, and immunogenicity of recombinant baculoviruses displaying MTB32C and MTB39A fusion antigenic proteins. The results show that this recombinant baculovirus is able to induce both humoral and cellular effects in mice and calves. In addition, it represents a potential vaccine candidate against MTB32C and MTB39A.

## Figures and Tables

**Table 1:** Antigens selected in this experiment.

Protein	NCBI Registration Number	Features/Functions	Amino Acid Lengths
MTB39A	NC_000962.3	PE/PPE protein family	391 aa
MTB32C	MTCI418B.07	Metabolism	132 aa

**Table 2:** Vaccination strategy in mice.

Group	Immunization Time Points	Dose
PBS	0, 14, 28 d	100 μL
rvAc-dual	0, 14, 28 d	10^7^ PFU
BCG	0, 14, 28 d	5 × 10^4^ CFU
rvAc-71cA	0, 14, 28 d	10^7^ PFU
rvAc-MTB39A	0, 14, 28 d	10^7^ PFU

## Data Availability

The original contributions presented in the study are included in the article/Supplementary Materials, further inquiries can be directed to the corresponding authors.

## Supplementary Materials

The following supporting information can be downloaded at https://www.mdpi.com/article/10.3390/ijms26020797/s1.
