# Supplementary material for: Development of a Vaccine Candidate Based on Surface-Displayed Particles of Mycobacterium tuberculosis from the MTB39A Protein
Source: Int J Mol Sci. 2025 Jan 18;26(2):797. doi: 10.3390/ijms26020797 (PMC11766116; doi:10.3390/ijms26020797)
Supplement: Supplementary file 1 [file ijms-26-00797-s001.zip › ijms-3355972-supplementary.pdf]

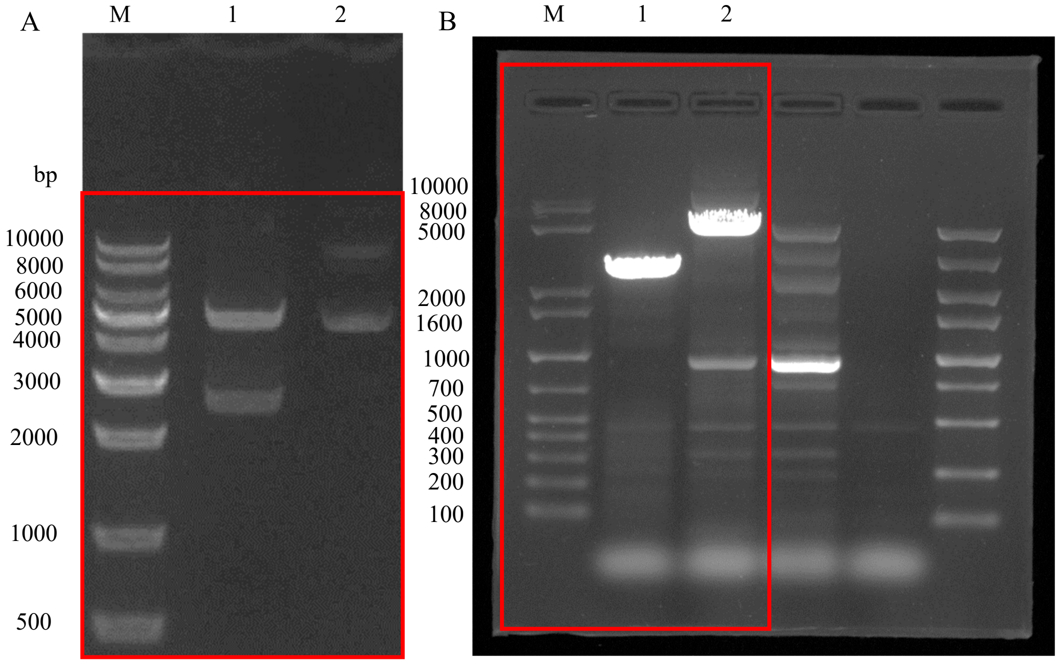

Supplementary Figure S1: (A) Uncropped Double digest of the recombinant vector pFastBac dual-71CA-mCherry.M: KB Ladder; 1: pFastBac dual-71cA-mCherry/*Bam*HI-*Hin*dIII(5238 bp, 2589bp); 2: the recombinant vector plasmid (B) PCR identification of recombinant bacmid-71CA-mCherry was not cropped. M: 1kb plus marker; 1: the pFastBac dual empty vector; 2: Bacmid-71CA-mCherry(5149 bp).

The red box represents the cropped part in the figure

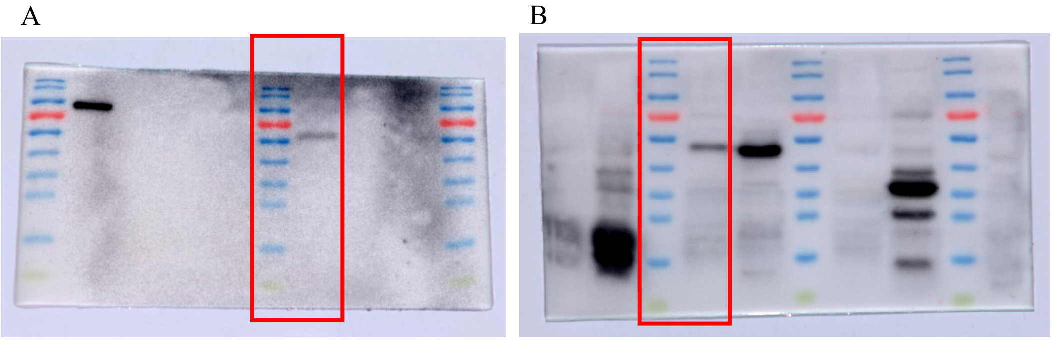

Supplementary Figure S2: Uncropped image Western blot identification of recombinant baculovirus surface-display particles. (A) M: 10-180 kDa; 1: Western blot identification of rvAc-71cA (63.5 kDa). (B) M: 10-180 kDa; 1: Western blot identification of rvAc-MTB39A (50.5 kDa).

The red box represents the cropped part in the figure.
